# Supplementary material for: The search for sexually antagonistic genes: Practical insights from studies of local adaptation and statistical genomics
Source: Evol Lett. 2020 Aug 31;4(5):398–415. doi: 10.1002/evl3.192 (PMC7523564; doi:10.1002/evl3.192)
Supplement: Supplementary file 1 — Figure S1. Permuted versus observed F^ST for simulated data. Figure S2. Permuted versus observed F^ST for the flycatcher dataset. Figure S3. Permuted versus observed F^ST for the pipefish dataset. Figure S4. Permuted vs. observed F^ST for the human 1000 Genomes data. Figure S5. Human 1000 Genomes data with SNPs with low minor allele frequencies (MAFs) included in the analysis. Appendix A. Distribution of FST estimates. Table S1. Sex‐specific relative fitness of genotypes of a biallelic locus with additive fitness effects in each sex. Appendix B. Sex‐specific allele frequencies and FIS estimates. Appendix C. Case‐control GWAS and the Log‐Odds Ratio. Figure S6. Probability that L. ⁄SE for an additive SA locus. Figure S7. Theoretical signal of multilocus SA polymorphism: the ratio of observed versus permuted absolute log odds ratio estimates within 100 quantiles of the theoretical null of L. ⁄SE. Appendix D. Comparison of null model distributions for different metrics allele frequency differentiation between sexes. [file EVL3-4-398-s001.pdf]

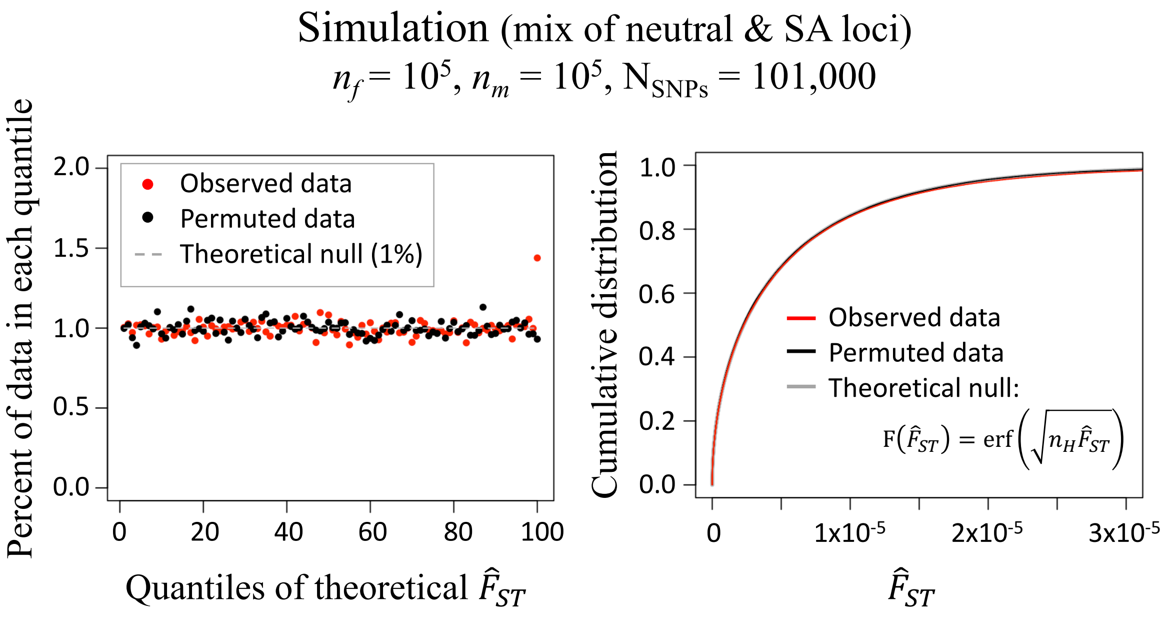

Figure S1. Permuted vs. observed $\hat{F}_{ST}$ for simulated data. Details follow those described in Fig. 2 of the main text.

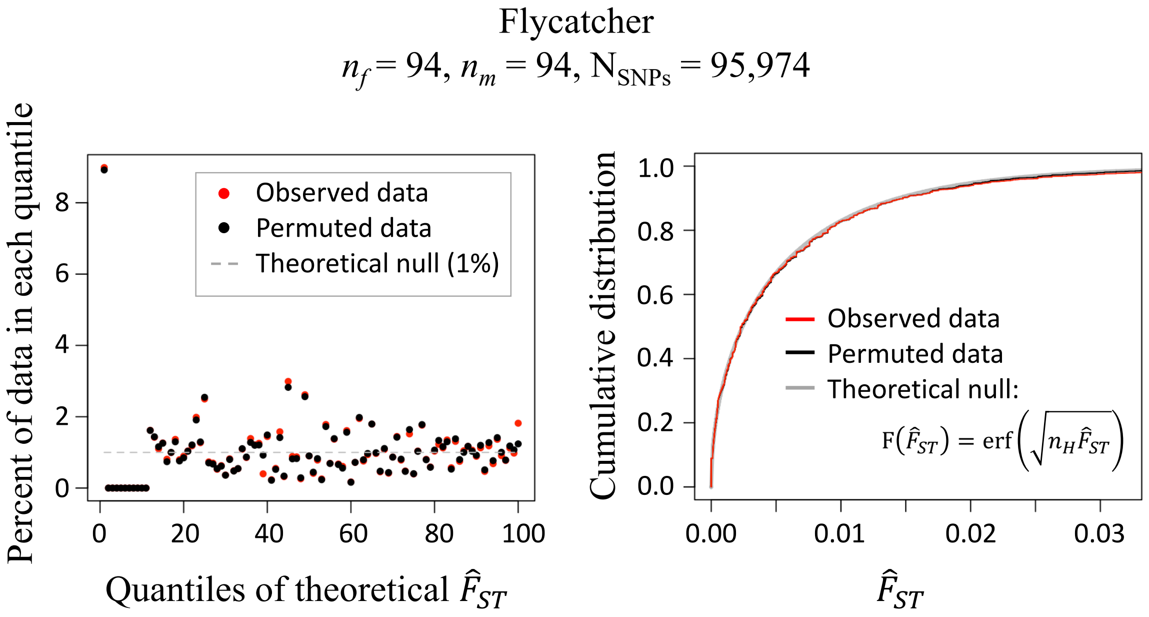

Figure S2. Permuted vs. observed $\hat{F}_{ST}$ for the flycatcher dataset. Details of the analysis follow those described in Fig. 2 of the main text.

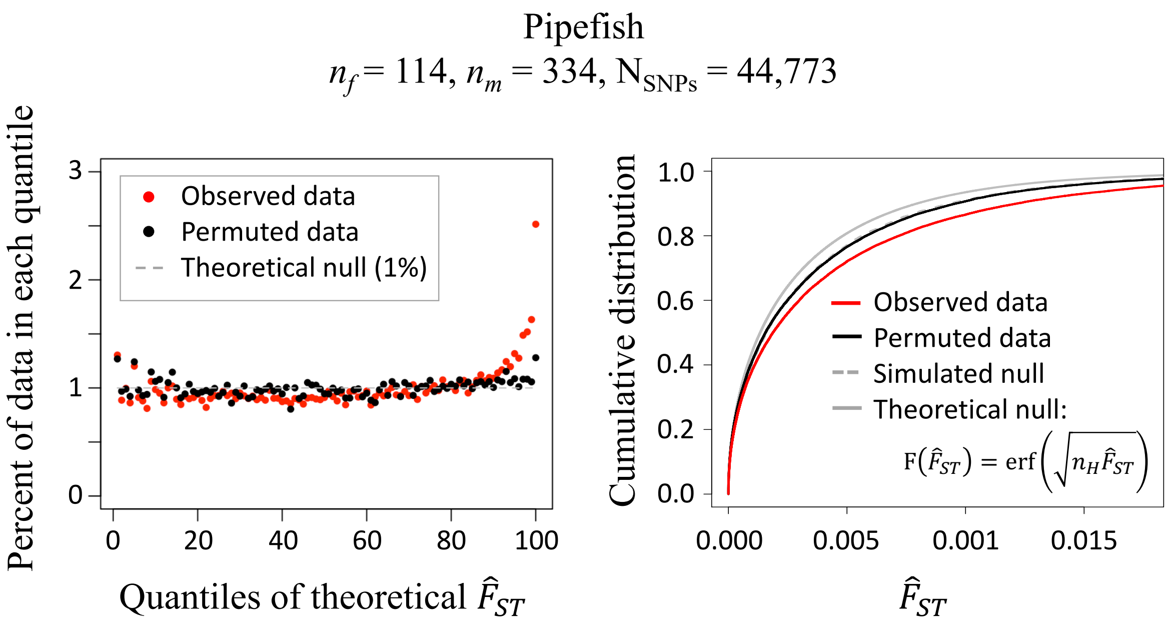

Figure S3. Permuted vs. observed $\hat{F}_{ST}$ for the pipefish dataset. Details of the analysis follow those described in Fig. 2 of the main text. Note that there are two null distributions in the right-hand panel. One is the idealized theoretical null distribution (the solid grey curve) which would apply if there was no variation in sample sizes across SNPs (*i.e.*, it requires that n_H is roughly constant across polymorphic sites in the genome). The idealized null model is inappropriate for the pipefish dataset due to missing data leading to sample size variation among sites. The simulated null accounts for missing data by simulating $\hat{F}_{ST}$ per site from a null distribution with site-specific n_H.

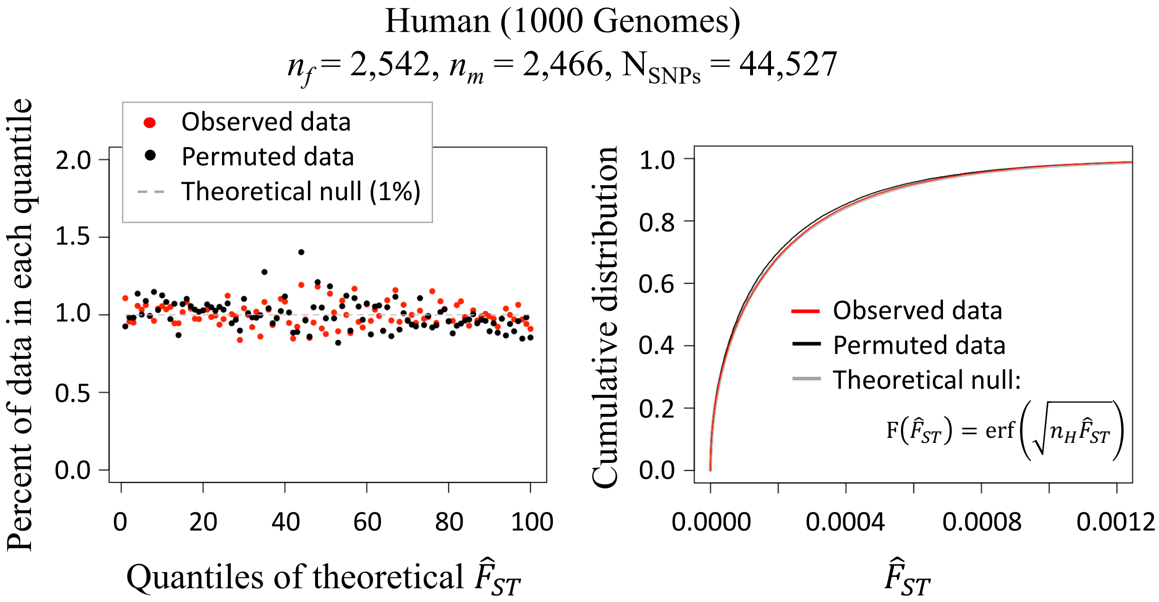

Figure S4. Permuted vs. observed $\hat{F}_{ST}$ for the human 1000 Genomes dataset. Details of the analysis follow those described in Fig. 2 of the main text.

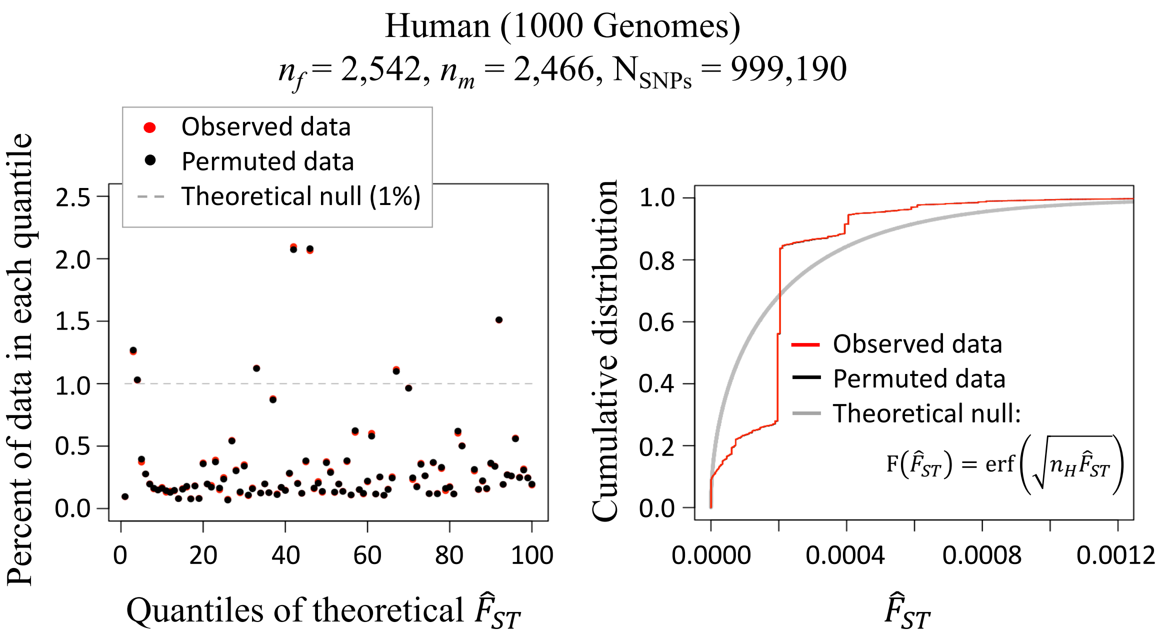

Figure S5: Human 1000 Genomes data with SNPs with low minor allele frequencies (MAFs) included in the analysis. All else is the same as Fig. S4.

Appendix A: Distribution of F_{ST} estimates

Following Cheng and Kirkpatrick (2016), between-sex F_{ST} for a bi-allelic locus is defined as:

$$F_{ST} = \frac{\left(p_f - p_m\right)^2}{4\bar{p}(1-\bar{p})}$$

where p_f and p_m are the female and male population frequencies of the focal allele at the locus, and $\bar{p}$ is the sex-averaged allele frequency, $\bar{p} = \left(p_f + p_m\right)/2$. The above expression applies to the entire population. Define F_{ST} estimated from allele frequencies in a sample of gene sequences as:

$$\hat{F}_{ST} = \frac{\left(\hat{p}_f - \hat{p}_m\right)^2}{4\hat{p}(1-\hat{p})}$$

(see Nei 1973), where $\hat{p}_f$ and $\hat{p}_m$ are the female and male frequencies calculated from the sample of sequences, and $\hat{p} = \left(\hat{p}_f + \hat{p}_m\right)/2$. The subsequent analysis is based on this simple estimator of between-sex F_{ST}. We note that several other F_{ST} estimators are widely used in the empirical literature (Bhatia et al 2013; Gammerdinger et al. 2020), and return to these alternative estimators further below.

Suppose that, at birth, the focal allele at a given locus has an overall population frequency of p in both sexes (the other allele at the locus has a frequency of $1-p$). Although genetic drift can lead to different allele frequencies between the sexes at birth, we assume that the population size is sufficiently large that such differences are negligible. As long as the sample size of sequenced individuals is large and the strength of selection at the locus is not extreme, then the allele frequencies in the panel of sequenced adults will be similar to the allele frequencies at birth in the population. We can therefore approximate F_{ST} from the sample of sequences using a Taylor Series expansion around $\hat{p}_f = \hat{p}_m = p$:

$$\begin{aligned}\hat{F}_{ST} = \left(\hat{p}_f - p\right)\frac{\partial \hat{F}_{ST}(\hat{p}_i = p)}{\partial \hat{p}_f} + (\hat{p}_m - p)\frac{\partial \hat{F}_{ST}(\hat{p}_i = p)}{\partial \hat{p}_m} \\ + \frac{1}{2}\Bigg[\left(\hat{p}_f - p\right)^2 \frac{\partial^2 \hat{F}_{ST}(\hat{p}_i = p)}{\partial {\hat{p}_f}^2} + (\hat{p}_m - p)^2 \frac{\partial^2 \hat{F}_{ST}(\hat{p}_i = p)}{\partial {\hat{p}_m}^2} \\ + 2\left(\hat{p}_f - p\right)(\hat{p}_m - p)\frac{\partial^2 \hat{F}_{ST}(\hat{p}_i = p)}{\partial \hat{p}_f \partial \hat{p}_m}\Bigg] + O[(\hat{p}_i - p)^3] \approx \frac{\left(\hat{p}_f - \hat{p}_m\right)^2}{4p(1-p)}\end{aligned}$$

with the final approximation dropping third-order and higher terms of $\hat{p}_i - p$.

Given the overall allele frequencies of p_f and p_m in adult females and males of the population, then $n_f \hat{p}_f$ and $n_m \hat{p}_m$ from samples of gene sequences will be independent, binomially distributed random variables: $\hat{p}_i \sim B(n_i, p_i)/n_i$, where n_i is the number of sequences derived from members of the ith sex. With sufficiently large n_i, the distribution of $\hat{p}_i$ will be approximately normal, $\hat{p}_i \sim N\left(n_i p_i, n_i p_i (1-p_i)\right)/n_i$, leading to:

$$\hat{p}_f - \hat{p}_m \sim N\left((p_f - p_m), \left(\frac{p_f(1-p_f)}{n_f} + \frac{p_m(1-p_m)}{n_m} \right) \right)$$

Using the normal approximation, $\hat{F}_{ST}$ simplifies to:

$$\hat{F}_{ST} \approx \frac{\left(\frac{p_f(1-p_f)}{n_f} + \frac{p_m(1-p_m)}{n_m} \right)}{4p(1-p)} \left(\frac{\hat{p}_f - \hat{p}_m}{\sqrt{\frac{p_f(1-p_f)}{n_f} + \frac{p_m(1-p_m)}{n_m}}} \right)^2$$
$$= \frac{n_m p_f(1-p_f) + n_f p_m(1-p_m)}{4 n_f n_m p(1-p)} X$$

where X is a non-central chi-squared distributed random variable with one degree of freedom, and a mean and variance of $\mathrm{E}[X] = 1 + (p_f - p_m)^2 \left(\frac{p_f(1-p_f)}{n_f} + \frac{p_m(1-p_m)}{n_m} \right)^{-1}$ and $\mathrm{var}[X] = 2\left(1 + 2(p_f - p_m)^2 \left(\frac{p_f(1-p_f)}{n_f} + \frac{p_m(1-p_m)}{n_m} \right)^{-1} \right)$. The mean and variance for $\hat{F}_{ST}$ (respectively) are:

$$\mathrm{E}[\hat{F}_{ST}] \approx \frac{1}{4p(1-p)} \left(\frac{p_f(1-p_f)}{n_f} + \frac{p_m(1-p_m)}{n_m} + (p_f - p_m)^2 \right)$$

and

$$\mathrm{var}[\hat{F}_{ST}] \approx 2\left(\frac{1}{4p(1-p)} \right)^2 \left(\frac{p_f(1-p_f)}{n_f} + \frac{p_m(1-p_m)}{n_m} \right) \left(\frac{p_f(1-p_f)}{n_f} + \frac{p_m(1-p_m)}{n_m} + 2(p_f - p_m)^2 \right)$$

For a locus with no sex differences in selection ($p \approx p_f \approx p_m$), $\hat{F}_{ST}$ reduces to:

$$\hat{F}_{ST} \approx \frac{n_m + n_f}{4 n_f n_m} X_0 = \frac{1}{2 n_H} X_0$$

where X_0 is a chi-squared random variable with one degree of freedom, and $n_H = 2/(1/n_f + 1/n_m)$ is the harmonic mean of the sex-specific sample sizes. The mean and variance for $\hat{F}_{ST}$ become:

$$\mathrm{E}[\hat{F}_{ST}] \approx \frac{n_m + n_f}{4 n_f n_m} = \frac{1}{2 n_H}$$

and

$$\mathrm{var}[\hat{F}_{ST}] \approx \frac{1}{8}\left(\frac{n_m + n_f}{n_f n_m}\right)^2 = \frac{1}{2n_H^2}$$

Under the null model, the variance of $\hat{F}_{ST}$ values divided by the square of the mean is:

$$\frac{\mathrm{var}[\hat{F}_{ST}]}{\mathrm{E}[\hat{F}_{ST}]^2} \approx 2$$

which is identical to the ratio for a recently subdivided pair of populations (Lewontin and Krakauer 1973; Nei and Chakravarti 1977).

For sufficiently large n_H, the median $\hat{F}_{ST}$ for neutral loci is approximately:

$$\mathrm{med}[\hat{F}_{ST}] \approx \frac{1}{2n_H}\left(\frac{7}{9}\right)^3 \approx \frac{0.47}{2n_H}$$

Since the distribution of $\hat{F}_{ST}$ for neutral loci is a simple linear function of chi-squared random variables, we can define the cumulative distribution of neutral $\hat{F}_{ST}$ using known properties of the chi-squared distribution. The cumulative distribution function for the chi-squared distribution with one degree of freedom is:

$$F(\hat{F}_{ST}) = \mathrm{erf}\left(\sqrt{n_H \hat{F}_{ST}}\right)$$

where $\mathrm{erf}(x)$ refers to the error function.

Effects of selection on allele frequency divergence between sexes

As in Box 1, supposing that selection is strong relative to genetic drift, then the allele frequency in the *i*th sex, following selection, will be:

$$p_i = p + s_i p(1-p)\big(h_i + p(1-2h_i)\big) + O(s_i^2)$$

where s_i and h_i are the selection and dominance coefficients for the focal allele in the *i*th sex. Under weak selection ($|s_i| << 1$), the first-order approximation (*i.e.*, neglecting $O(s_i^2)$) provides a good approximation for the allele frequencies, per sex, following selection.

The first-order approximation can, however, prove inaccurate in scenarios involving strong selection (*i.e.*, $|s_f|$, $|s_m| > 0.1$), including cases of SA selection with which we are concerned. For the case of arbitrarily strong SA selection, assume that the fitness effects of SA alleles are additive in each sex (*i.e.*, $h_f = h_m = ½$), and the population is at polymorphic equilibrium ($p_f + p_m = 2p$). For this scenario, we can calculate exact allele frequencies per sex, following selection.

Assume that the *A* allele is female-beneficial and the *a* allele is male-beneficial (*i.e.*, $s_f > 0 > s_m$ in Box 1 of the main text). Following Kidwell et al. (1977), we first re-parameterize the general fitness model by substituting $s_m = -|s_m|$, and $t_f = s_f/(1 + s_f)$ so that relative fitnesses in each sex are scaled against the best genotype in that sex (Table S1). With no dominance ($h_f =$

h_m = ½), we obtain a symmetric SA parameterization, where $|s_m|$ and t_f represent the fitness costs to females and males (respectively) of carrying the "wrong" allele (Table S1). Both selection parameters are constrained to be between zero and one ($0 < |s_m|, t_f < 1$), and $t_f = |s_m|$ represents the special case where the strength of selection is equal between the sexes.

Table S1. Sex-specific relative fitness of genotypes of a bi-allelic locus with additive fitness effects in each sex (no dominance; $h_i = ½$)

	**Genotype**		
	AA	***Aa***	***aa***
**Original parameterization (Box 1)**			
female relative fitness	$1 + s_f$	$1 + s_f/2$	1
male relative fitness	$1 + s_m$	$1 + s_m/2$	1
**Re-parameterization for a SA locus ($s_f > 0 > s_m$)**			
female relative fitness	1	$1 - t_f/2$	$1 - t_f$
male relative fitness	$1 - \|s_m\|$	$1 - \|s_m\|/2$	1

Given the reparameterization in Table S1, previous theory (see Connallon and Jordan 2016) has shown that the equilibrium *A* allele frequency of a polymorphic SA locus will be:

$$p = \frac{t_f - |s_m| + t_f|s_m|}{2t_f|s_m|}$$

and the equilibrium frequency difference between breeding females and males is:

$$p_f - p_m = \frac{2(1 - p|s_m|)}{|s_m|}\left(\sqrt{1 + \frac{t_f|s_m|p(1-p)}{(1 - p|s_m|)(1 - (1-p)t_f)}} - 1\right)$$

With an intermediate equilibrium frequency (p = ½, which requires that $t_f = |s_m|$), the expected $\hat{F}_{ST}$ for the locus is:

$$\mathrm{E}[\hat{F}_{ST}] = \frac{1}{2n_H} + \frac{(p_f - p_m)^2}{4p(1-p)}\left(1 - \frac{1}{2n_H}\right)$$

Given the sampling distribution of $\hat{F}_{ST}$ (Box 2; see above), when should we expect $\hat{F}_{ST}$ for a SA SNP to reliably reside within the tail of the null distribution for $\hat{F}_{ST}$? Consider the following approximations for when such outcomes are likely. Assuming that each SA locus has additive fitness effects (*i.e.*, $h_f = h_m$ = ½ and $s_f > 0 > s_m$ in Box 1), and each has evolved to an intermediate equilibrium frequency (p = ½ and $|s_m| = s_f/(1 + s_f)$, where $|s_m|$ represents the fitness cost to males of inheriting *AA* and $s_f/(1 + s_f)$ is the cost to females of inheriting *aa*; see Connallon and Jordan 2016), $\hat{F}_{ST}$ for a selected locus will have a relatively high probability of residing within the tail of the null when its expected value, $\mathrm{E}[\hat{F}_{ST}|SA]$, is greater than the percentile that is used to define the tail of the null. Following Box 2, the kth percentile of the null distribution for $\hat{F}_{ST}$ is:

$$\hat{F}_{ST(k)} = \frac{\left[\text{erf}^{-1}\left(\frac{k}{100}\right)\right]^2}{n_H}$$

where $\text{erf}^{-1}(x)$ refers to the inverse error function. Given the stated assumptions, the expected $\hat{F}_{ST}$ for an SA locus will be greater than the kth percentile of the null (*i.e.*, $\text{E}\left[\hat{F}_{ST}|SA\right] > \hat{F}_{ST(k)}$), when $|s_m|$ exceeds the following threshold:

$$s_{\min} = \frac{4\sqrt{\dfrac{2\left[\text{erf}^{-1}\left(\frac{k}{100}\right)\right]^2 - 1}{2n_H - 1}}}{1 - \dfrac{2\left[\text{erf}^{-1}\left(\frac{k}{100}\right)\right]^2 - 1}{2n_H - 1} + 2\sqrt{\dfrac{2\left[\text{erf}^{-1}\left(\frac{k}{100}\right)\right]^2 - 1}{2n_H - 1}}}$$

(see Fig. 1A), where $s_{\min}$ represents the minimum strength of selection (*i.e.*, the minimum cost of inheriting the "wrong" SA allele, per sex) that satisfies $\text{E}\left[\hat{F}_{ST}|SA\right] \geq \hat{F}_{ST(k)}$. Simulations over a range of sample sizes show that $\hat{F}_{ST}$ for a SA locus at equilibrium with $|s_m| = s_f/(1 + s_f) = s_{\min}$ has a ~40-50% probability of exceeding the 99th percentile of the null. For the exact probability that $\hat{F}_{ST}$ for a SA SNP exceeds the 95th percentile of the $\hat{F}_{ST}$ null distribution, see Fig. 1B of the main manuscript and Appendix D, below. Even stronger selection is required to satisfy the condition $\text{E}\left[\hat{F}_{ST}|SA\right] > \hat{F}_{ST(k)}$ when one SA allele at the locus is rarer than the other ($p \neq ½$; see Appendix D) and/or when dominance is reversed between sexes (*i.e.*, $h_f > ½ > h_m$; see Connallon and Jordan 2016).

Other F_{ST} estimators

The F_{ST} estimator presented above and in Box 2 is a biased estimator with an average that is greater than zero under the null model in which there are no allele frequency differences between the sexes in the population as whole (*i.e.*, $\hat{F}_{ST}$, as defined above has an expectation greater than zero when $p_f = p_m$ in the population). Since alternative estimators are often used in empirical studies of population or sex differentiation, it is worth exploring the distribution of $\hat{F}_{ST}$ based on alternative estimators.

The most widely used estimator, Weir-Cockerham's F_{ST} (Weir and Cockerham 1984; Bhatia et al 2013), is defined as:

$$\hat{F}_{ST} = 1 - \frac{\frac{n_H}{n_f + n_m - 2}\left[n_f\hat{p}_f(1 - \hat{p}_f) + n_m\hat{p}_m(1 - \hat{p}_m)\right]}{\frac{n_H}{2}\left(\hat{p}_f - \hat{p}_m\right)^2 + \frac{n_H - 1}{n_f + n_m - 2}\left[n_f\hat{p}_f(1 - \hat{p}_f) + n_m\hat{p}_m(1 - \hat{p}_m)\right]}$$

where $n_H = 2\left(1/n_f + 1/n_m\right)^{-1}$. Assuming large populations and (at most) moderate selection, then the estimator can be approximated using a Taylor series expansion around $\hat{p}_f = \hat{p}_m = p$, where p is the allele frequency at birth:

$$\hat{F}_{ST} = \frac{1}{1-n_H} + \frac{1}{2}\left[\left(\hat{p}_f - p\right)^2 \frac{\partial^2 F_{ST}(\hat{p}_i = p)}{\partial \hat{p}_f^{\,2}} + (\hat{p}_m - p)^2 \frac{\partial^2 F_{ST}(\hat{p}_i = p)}{\partial \hat{p}_m^{\,2}} \right.$$
$$\left. + 2\left(\hat{p}_f - p\right)(\hat{p}_m - p)\frac{\partial^2 F_{ST}(\hat{p}_i = p)}{\partial \hat{p}_f \partial \hat{p}_m}\right] + O((\hat{p}_i - p)^3)$$
$$\approx \frac{1}{1-n_H}$$
$$+ \left(\frac{n_H}{n_H - 1}\right)^2 \left(1 - \frac{2}{n_f + n_m}\right) \frac{\left(\hat{p}_f - p\right)^2 + (\hat{p}_m - p)^2 - 2\left(\hat{p}_f - p\right)(\hat{p}_m - p)}{2p(1-p)}$$
$$= \frac{1}{1-n_H} + \left(\frac{n_H}{n_H - 1}\right)^2 \left(1 - \frac{2}{n_f + n_m}\right) \frac{\left(\hat{p}_f - \hat{p}_m\right)^2}{2p(1-p)}$$

Under the null model, we have (approximately):

$$\hat{p}_f - \hat{p}_m \sim N\left(0, \frac{2p(1-p)}{n_H}\right)$$

and

$$\hat{F}_{ST} \approx \frac{1}{1-n_H} + \frac{n_H}{(1-n_H)^2}\left(1 - \frac{2}{n_f + n_m}\right) X_0$$

where X_0 is a chi-squared random variable with one degree of freedom (as above). For large n_H, we can approximate the F_{ST} estimator as:

$$\hat{F}_{ST} \approx \frac{1}{n_H}(X_0 - 1)$$

which gives a mean and variance of:

$$\mathrm{E}\left[\hat{F}_{ST}\right] \approx 0$$

$$\mathrm{var}\left[\hat{F}_{ST}\right] \approx \frac{2}{n_H^2}$$

The mean is now centred at zero, which shows that the Weir and Cockerham estimator is asymptotically unbiased. Nevertheless, the sampling variance remains inversely proportional to the square of the sample size and will therefore show a similar magnitude of sampling variance as our focal estimator: $\hat{F}_{ST} = \left(\hat{p}_f - \hat{p}_m\right)^2 / 4\hat{p}(1-\hat{p})$.

Another commonly used estimator is that of Hudson et al. (1992), which for our case is expressed as:

$$\hat{F}_{ST} = \frac{\left(\hat{p}_f - \hat{p}_m\right)^2 - \frac{\hat{p}_f\left(1-\hat{p}_f\right)}{n_f - 1} - \frac{\hat{p}_m(1-\hat{p}_m)}{n_m - 1}}{\hat{p}_f(1-\hat{p}_m) + \hat{p}_m\left(1-\hat{p}_f\right)}$$

(see Bhatia et al 2013; Gammerdinger et al. 2020). The estimators of Hudson et al. and Weir and Cockerham are identical when the sample sizes of female- and male-derived sequences are equal ($n_f = n_m$), and in practice is approximately the same as Weir and Cockerham's estimator under the null model ($p_f = p_m$) with very large sample sizes of female and male sequences.

Genic F_{ST} estimates

Booker et al. (2020) recently emphasized that the distribution of F_{ST} estimated from concatenated sequences will strongly depend on the degree of linkage between polymorphic sites in the sequence. Specifically, the chi-square distribution of $\hat{F}_{ST}$ under the null remains applicable when there is no recombination between sites in the concatenated sequence. With recombination, the expected $\hat{F}_{ST}$ remains the same, whereas the variance declines. This effect of concatenation applies to the context of F_{ST} between sexes, as we illustrate below. As such, we advocate using non-concatenated estimates to evaluate fits between observed and theoretical distributions for $\hat{F}_{ST}$.

Using our original F_{ST} estimator, between-sex F_{ST} for a single neutral locus is:

$$\hat{F}_{ST} = \frac{(\hat{p}_f - \hat{p}_m)^2}{4\hat{p}(1-\hat{p})} = \frac{X_0}{2n_H}$$

where, as before, X_0 is a chi-square random variable with one degree of freedom and n_H is the harmonic mean sample size of female and male gene sequences.

Now define a concatenated genic F_{ST} as the average across k polymorphic sites in a given gene. Assuming the k sites are neutral, we have:

$$\hat{F}_{ST} = \frac{1}{k}\sum_{i=1}^{k} \hat{F}_{ST,i} = \frac{1}{k}\sum_{i=1}^{k} \frac{X_i}{2n_{H,i}}$$

where subscripts refer to the ith polymorphic site in the gene.

If the k sites are independently segregating and sequence sample sizes are identical across sites ($n_{H,i} = n_H$), then genic $\hat{F}_{ST}$ simplifies to:

$$\hat{F}_{ST} = \frac{1}{2kn_H}\sum_{i=1}^{k} X_i \sim \frac{\chi_k^2}{2kn_H}$$

where $\sum_{i=1}^{k} X_i \sim \chi_k^2$ is a chi-square random variable with k degrees of freedom. The mean and variance for the estimated genic $\hat{F}_{ST}$ are:

$$\mathrm{E}[\hat{F}_{ST}] = \frac{1}{2n_H}$$

and

$$\mathrm{var}\left[\hat{F}_{ST}\right] = \frac{1}{2kn_H^2}$$

We see that under the null model, the expected $\hat{F}_{ST}$ for a SNP and for a genic region will be the same, whereas the former has a k-fold greater variance than the latter. Outliers are, therefore, more likely to arise in genes with lower numbers of polymorphisms or nonindependence (non-zero LD) between polymorphic sites.

Appendix B: Sex-specific allele frequencies and F_{IS} estimates

We assume an outbred population with random mating between the breeding adults of each sex. Let p_f and p_m represent the allele frequencies in breeding females and males, as in Appendix A. Following Kasimatis et al. (2019), we define F_{IS} as the degree of enrichment in heterozygosity among offspring of the population, which is a consequence of the allele frequency differences between breeding adults of the prior generation. Defining p as the frequency of the focal allele in offspring of the breeding adults (so that $2p = p_f + p_m$), we have:

$$F_{IS} = \frac{p_f(1-p_m) + p_m(1-p_f)}{2p(1-p)} - 1 = \frac{p_f(1-p_m) + p_m(1-p_f) - 2p(1-p)}{2p(1-p)}$$
$$= \frac{2\left(p - p_f p_m - p(1-p)\right)}{2p(1-p)} = \frac{\left(p_f - p_m\right)^2}{4p(1-p)}$$

Note that the final expression is identical to F_{ST} between sexes for the population of breeding females and males of the prior generation (see above). The deviation from Hardy-Weinberg Equilibrium (HWE) can be quantified using a disequilibrium coefficient, D_A (see Weir 1996, p. 94), in which case F_{IS} becomes:

$$F_{IS} = \frac{P_{Aa}}{2p(1-p)} - 1 = \frac{2p(1-p) - 2D_A}{2p(1-p)} - 1 = -\frac{D_A}{p(1-p)}$$

where P_{Aa} is the frequency of heterozygotes in the population, and $D_A = (p(1-p) - P_{Aa}/2)$ quantifies the HWE deviation. Note that $D_A > 0$ implies a deficit of heterozygotes relative to HWE (as expected under inbreeding or population subdivision), whereas $D_A < 0$ implies an excess of heterozygotes (as expected when there are allele frequency differences between the sexes).

The null distribution of F_{IS}

With large genotype samples (large n, where n refers to the number of diploid offspring that are sequenced), empirical estimates of D_A, denoted $\widehat{D}_A$, will be approximately normal (Weir 1996, p. 95), with expectation and variance:

$$\mathrm{E}\left[\widehat{D}_A\right] = D_A - \frac{p(1-p) + D_A}{2n}$$

$$\mathrm{var}\left[\widehat{D}_A\right] = \frac{p^2(1-p)^2 + (1-2p)^2 D_A - D_A^2}{n}$$

Consequently, $\left(\widehat{D}_A - \mathrm{E}\left[\widehat{D}_A\right]\right)/\sqrt{\mathrm{var}\left[\widehat{D}_A\right]}$ will follow a standard normal distribution. If the population really is at HWE (as under the null model), and the sample size is sufficiently large so that the variance in the test statistic can be approximated using $\mathrm{var}\left[\widehat{D}_A\right] = \frac{\hat{p}^2(1-\hat{p})^2}{n}$, then:

$$\frac{\widehat{D}_A}{\sqrt{\mathrm{var}\left[\widehat{D}_A\right]}} = \frac{\widehat{D}_A\sqrt{n}}{\hat{p}(1-\hat{p})} \sim N\left(-\frac{1}{2\sqrt{n}}, 1\right)$$

From this, we see that the estimate of F_{IS} (denoted $\hat{F}_{IS}$) is approximately normal with mean $\mathrm{E}[\hat{F}_{IS}] = 1/2n$ and variance $\mathrm{var}[\hat{F}_{IS}] = 1/n$, *i.e.*:

$$\hat{F}_{IS} = -\frac{\hat{D}_A}{\hat{p}(1-\hat{p})} = -\frac{1}{\sqrt{n}}\left(\frac{\hat{D}_A\sqrt{n}}{\hat{p}(1-\hat{p})}\right) \sim N\left(\frac{1}{2n}, \frac{1}{n}\right)$$

Simulations of the mean and variance for $\hat{F}_{IS}$ under the null model for 10^5 polymorphic loci with minor allele frequencies (MAF) greater than 0.05, are plotted below (lines show the analytical predictions, which match up well against the simulated data; note that the *y*-axis is in log scale).

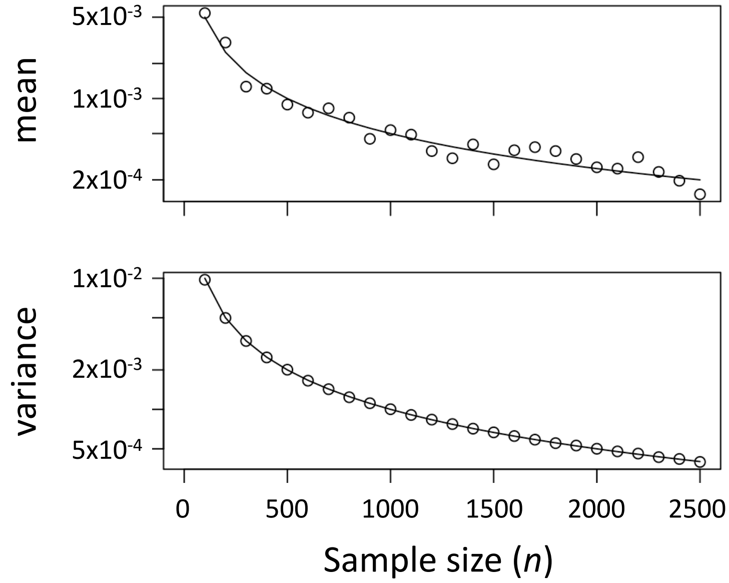

F

With sex differences in selection, the frequency of heterozygotes at birth will be:

$$p_f(1-p_m) + p_m(1-p_f) = 2p(1-p) + \frac{1}{2}(p_f - p_m)^2$$

where $p = (p_f + p_m)/2$. The HWE deviation in the population becomes:

$$D_A = -\frac{1}{4}(p_f - p_m)^2$$

Assuming that the sample size is large and the HWE deviation is small ($|D_A| << 1$), so that terms of $O(D_A^2, D_A n^{-1})$ can be neglected, the distribution of $\hat{F}_{IS}$ converges, approximately, to:

$$\hat{F}_{IS} = -\frac{\hat{D}_A}{\hat{p}(1-\hat{p})} \sim N\left(\frac{1}{2n} - \frac{D_A}{p(1-p)}, \frac{1}{n}\right)$$

Substituting $D_A = -\frac{1}{4}(p_f - p_m)^2$, we obtain:

$$\hat{F}_{IS} = -\frac{\hat{D}_A}{\hat{p}(1-\hat{p})} \sim N\left(\frac{1}{2n} + \frac{\left(p_f - p_m\right)^2}{4p(1-p)}, \frac{1}{n}\right)$$

The kth percentile of the null distribution ($p_f = p_m$) is:

$$\hat{F}_{IS(k)} = \frac{1}{2n} + \sqrt{\frac{2}{n}}\text{erf}^{-1}\left(\frac{2k}{100} - 1\right)$$

Using the exact expressions for a polymorphic locus at equilibrium under additive SA selection (h_i = ½; see Appendix A), the minimum fitness cost that will cause $\text{E}\left[\hat{F}_{IS}\right]$ for a SA SNP to reach the kth quantile for the null model (*i.e.*, the value of $|s_m|$, assuming p = ½, that leads to $\text{E}\left[\hat{F}_{IS}\right] = \hat{F}_{IS(k)}$) is:

$$s_{\min} = \frac{4\sqrt{\sqrt{\frac{2}{n}}\text{erf}^{-1}\left(\frac{2k}{100} - 1\right)}}{1 - \sqrt{\frac{2}{n}}\text{erf}^{-1}\left(\frac{2k}{100} - 1\right) + 2\sqrt{\sqrt{\frac{2}{n}}\text{erf}^{-1}\left(\frac{2k}{100} - 1\right)}}$$

(see Fig. 1A).

Appendix C: Case-control GWAS and the Log-Odds Ratio

As described in the main text and highlighted in a recent study of SA selection in humans (Kasimatis et al. 2020 bioRχiv), case-control GWAS methods offer a promising alternative to fixation indices (F_{ST} and F_{IS}) for estimating and testing for statistical significance of between-sex allele frequency differences. The advantage of case-control GWAS methods relative to genome-wide scans of between-sex F_{ST} and F_{IS} is that they leverage a well-developed linear modelling framework to explicitly account for confounding factors such as population connectivity when testing for allele frequency differences between sexes. Broadly speaking, case-control GWAS rely on a logistic regression of allele frequency on *sex* (coded as a categorical variable) and other possible explanatory variables, and hypothesis testing is based on the log-odds ratio. Below, we briefly outline the use of the log-odds ratio to test for between-sex differences in allele frequency, illustrate the limits of statistical power when using this approach, and highlight comparisons with the $\hat{F}_{ST}$ statistic, which is presented in the main text.

We emphasize that our brief summary is not meant to be a comprehensive statistical review of odds-ratios, nor a review of case-control GWAS methods and their use to control for population structure and other confounding variables (see *e.g.*, Pirastu et al. 2020; Astle and Balding 2009; Price et al. 2010). We also note that there is a deep and still-evolving statistical literature on hypothesis testing in case-control methods that readers should be aware of (*e.g.*, Haldane 1956, Wang 2015).

The log-odds ratio ($\mathcal{L}$)

Given genome sequence data for a random sample of adult males and females, one can construct a 2 × 2 contingency table of allele counts in males and females for a given biallelic locus as:

	A	a	*Total*
Males	$n_{m,A}$	$n_{m,a}$	n_m
Females	$n_{f,A}$	$n_{f,a}$	n_f
Total	n_A	n_a	

where $n_{i,j}$ is the number of copies of the jth allele type in the sample of genes sequenced from the ith sex; as in our F_{ST} model n_f and n_m are the total sample sizes of genes sequenced in females and males, respectively. The allele frequency estimates from the samples are:

	A	a
Males	$\hat{p}_m$	$1-\hat{p}_m$
Females	$\hat{p}_f$	$1-\hat{p}_f$

where $\hat{p}_i = n_{i,A}/n_i$ with $n_i = n_{i,A} + n_{i,a}$. The sample log-odds ratio can be expressed as:

$$\hat{\mathcal{L}} = \log\left(\frac{\hat{p}_m(1-\hat{p}_f)}{\hat{p}_f(1-\hat{p}_m)}\right)$$

For large-sample sizes, $\hat{\mathcal{L}}$ is approximately normal with mean of $\mathrm{E}[\hat{\mathcal{L}}] = \log\left(\frac{p_m(1-p_f)}{p_f(1-p_m)}\right)$, where p_f and p_m refer to the true allele frequencies in the population, and a variance of $\mathrm{var}[\hat{\mathcal{L}}] = \frac{1}{n_{m,A}} + \frac{1}{n_{m,a}} + \frac{1}{n_{f,A}} + \frac{1}{n_{f,a}}$ (Woolf 1955; Section 14.6 in Bishop et al. 1975). We note that hypothesis testing relies heavily on this normal approximation, which breaks down for small sample sizes, and for loci with very low minor allele frequency (MAF) (see *e.g.*, Wang & Shan 2015).

The null distribution of $\hat{\mathcal{L}}$

With large genotype samples (large n_f and n_m), and assuming that there is no sex difference in selection (and thus no allele frequency difference between sexes in the population), empirical estimates of $\hat{\mathcal{L}}$ can be approximated by the Taylor series expansion:

$$\hat{\mathcal{L}} = \log\left(\frac{\hat{p}_m(1-\hat{p}_f)}{(1-\hat{p}_m)\hat{p}_f}\right) = -\frac{(\hat{p}_f - \hat{p}_m)}{\hat{p}(1-\hat{p})} + O\left[(\hat{p}_f - \hat{p}_m)^3\right]$$

Ignoring terms of $O\left[(\hat{p}_f - \hat{p}_m)^3\right]$, which are negligible with large sample sizes, $\hat{\mathcal{L}}$ is approximately normal with expectation and variance (respectively) of:

$$\mathrm{E}[\hat{\mathcal{L}}] = 0$$

$$\mathrm{var}[\hat{\mathcal{L}}] = \frac{1}{n_{m,A}} + \frac{1}{n_{m,a}} + \frac{1}{n_{f,A}} + \frac{1}{n_{f,a}} \approx \frac{2}{n_H\hat{p}(1-\hat{p})}$$

The scaled log-odds ratio, $\hat{\mathcal{L}}/\sqrt{\mathrm{var}[\hat{\mathcal{L}}]}$, follows a standard normal distribution, which provides a useful null distribution in the absence of sex-specific selection. The approximation breaks down when any of the allele counts are small, and reliable hypothesis testing therefore requires that the MAF is not too low, or that the sample size is large enough to ensure reasonably high counts of each allele type.

$\mathcal{L}$ under sex-specific selection

From Box 1 in the main text, the population frequency of the *A* allele within the *i*th sex, following sex-specific selection, will be:

$$p_i = p + s_i p(1-p)\big(h_i + p(1-2h_i)\big) + O(s_i^2)$$

where s_i and h_i are the selection and dominance coefficients for the focal allele in the *i*th sex. Analytic expressions for $\mathcal{L}$ under sex-specific selection quickly become unwieldy. We can, however, briefly illustrate the expected behavior of the distribution under sex-specific selection in the idealized scenario of intermediate initial allele frequency (*i.e.*, $p = ½$) and weak and additive sex-specific selection ($h_f = h_m = ½$; second-order terms $O(s_i^2)$ ignored). Under these simplifying assumptions, the distribution of $\hat{\mathcal{L}}$ is normal with mean and standard deviation:

$$\mathrm{E}[\hat{\mathcal{L}}] = \log\left[\frac{(4-s_f)(4+s_m)}{(4+s_f)(4-s_m)}\right]$$

$$SE = 8\sqrt{\frac{1}{n_f\left(16-s_f^2\right)}+\frac{1}{n_m\left(16-s_m^2\right)}}.$$

Simulations of the mean, standard error, and scaled log odds ratio for 10^4 loci conforming to the above assumptions show that the approximation works well for an illustrative scenario of SA selection ($s_m = 0.03$, $s_f = -0.03$) with equal sample sizes for both sexes (where i indexes sex and $n_m = n_f$), provided sample sizes are sufficiently large. Note that results for the null in the second panel are overlain by those for SA selection.

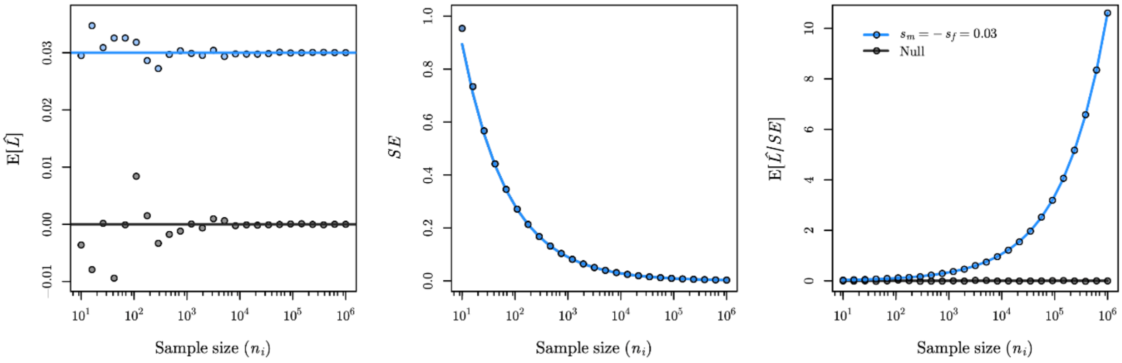

The expected distribution of the scaled sample log odds ratio will also be asymptotically normal, with

$$\frac{\hat{\mathcal{L}}}{SE} \sim \mathrm{N}\left(\frac{\log\left[\frac{(4-s_f)(4+s_m)}{(4+s_f)(4-s_m)}\right]}{8\sqrt{\frac{1}{n_f\left(16-s_f^2\right)}+\frac{1}{n_m\left(16-s_m^2\right)}}}, 1\right)$$

As noted above, the asymptotic properties of the sampling distribution of $\hat{\mathcal{L}}/SE$ break down when either MAFs are low, sample sizes are small, or both.

Statistical power to detect individual outlier loci

The probability that a SA locus resides within the tail of the null distribution depends on the allele frequencies at the locus, the strength of selection, and the sample size of individuals that are sequenced. The expected difference in allele frequency between the sexes, after selection, is maximized for a locus with intermediate allele frequencies and symmetric SA selection (p = ½, described above), representing a best-case scenario for detecting individual outlier loci. The robustness of the normal approximation for the scaled log odds ratio greatly simplifies calculation of statistical power to identify outlier loci under sex-specific selection. For our idealized scenario where $p_f = p_m = ½$ it is simply the area under $N\left(E\left[\frac{\hat{\mathcal{L}}}{SE}\right], 1\right)$ that falls above/below the α/2 and (1 – α)/2 quantiles of a standard normal distribution, $N(0,1)$. Below, we illustrate the probability that $\hat{\mathcal{L}}/SE$ reliably resides in the tail of the null distribution under the same parameter conditions shown for $\hat{F}_{ST}$ in Fig. 1B of the main text. Overall, the scaled log odds ratio provides similar statistical power to detect individual outlier loci as $\hat{F}_{ST}$. This final conclusion applies across the spectrum of MAF (see Appendix D, below).

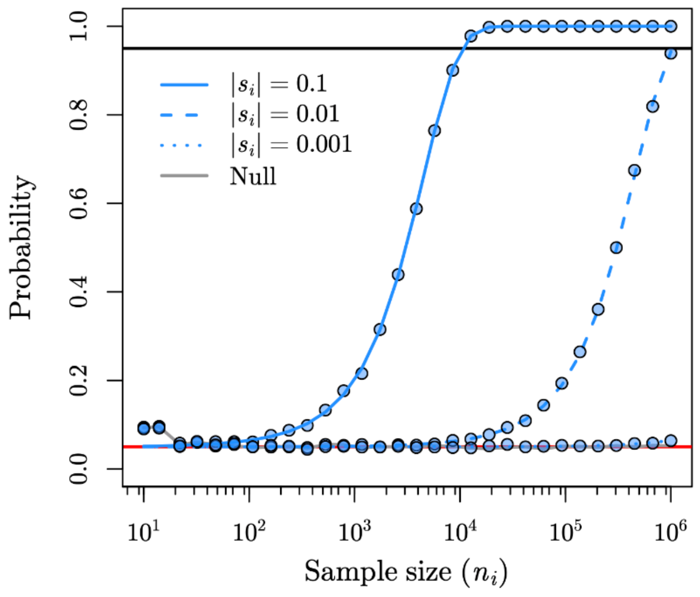

Figure S6 Probability that $\hat{\mathcal{L}}/SE$ for an additive SA locus (*i.e.*, $h_f = h_m = ½$) with intermediate equilibrium allele frequencies (p, q = 1/2) is in the top 2.5% tail of the null distribution $N(0,1)$. Sample sizes are assumed to be equal for the ith sex, and $|s_i|$ is the fitness cost of being homozygous for the "wrong" SA allele, where $i \in \{f,m\}$. Blue lines show the analytic approximation based on $N\left(E\left[\frac{\hat{\mathcal{L}}}{SE}\right], 1\right)$, points show the proportion of 10^4 simulated loci which fall in the upper and lower 2.5% tails of the null distribution. Red and black horizontal lines benchmark the 2.5% and 97.5% power thresholds.

Multi-locus signals of sex-specific selection using $|\hat{\mathcal{L}}/SE|$

Following identical logic as for $\hat{F}_{ST}$ in the main text, the full empirical distribution of the absolute value for $\hat{\mathcal{L}}/SE$ for autosomal SNPs may carry a cumulative signature of SA selection in the form of an inflated total number of observations in the upper quantiles of the observed $\hat{\mathcal{L}}/SE$ distribution relative to the number of observations predicted under the null. Below we illustrate the signal of enrichment of observations in the upper theoretical quantiles caused by SA selection using the ratio of observed versus permuted $\hat{\mathcal{L}}/SE$ for SNPs within 100 quantiles of the theoretical null of $\hat{\mathcal{L}}/SE$ using identical simulation methods as those presented in Fig. 2 in the main text (*i.e.*, the top panel of Figure 2). The scaled log odds ratio appears to provide a similar signal of SA selection to $\hat{F}_{ST}$, but is slightly less sensitive to shifts in the observed distribution due to SA selection (compare to Fig. 2 in the main text)

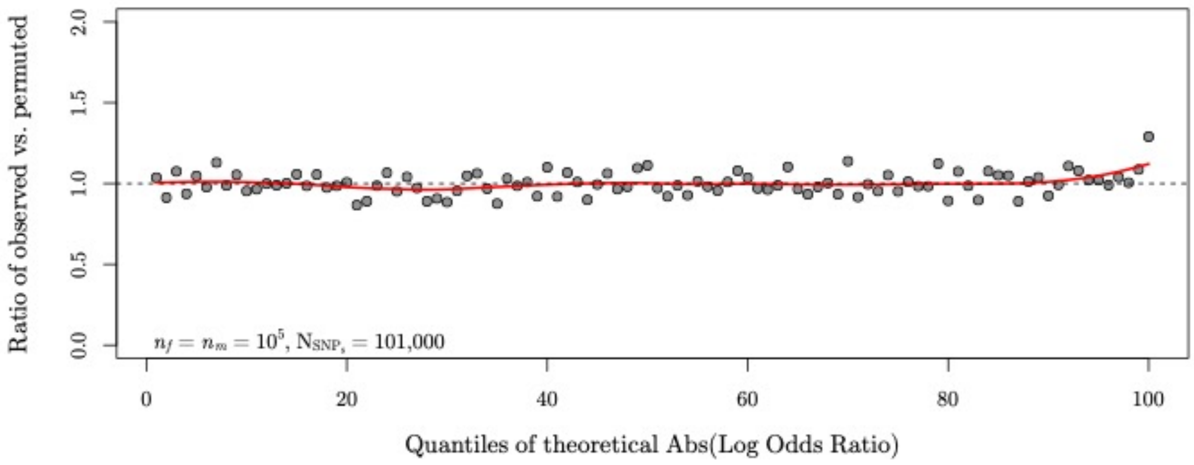

Figure S7. Theoretical signal of multi-locus SA polymorphism: the ratio of observed versus permuted absolute log odds ratio estimates within 100 quantiles of the theoretical null of $\hat{\mathcal{L}}/SE$. **Theoretical data:** $\hat{\mathcal{L}}/SE$ values were simulated for 10^5 neutrally evolving loci with MAF above 5% in the dataset, and for 10^3 loci responding to SA selection prior to sampling of gene sequences (each SA-responding locus had a SA fitness effect or was in perfect linkage disequilibrium with a SA locus). SA-responding loci had intermediate allele frequencies in the population (p = ½). SA selection coefficients were drawn from an exponential distribution with mean of s_{avg} = 0.03, and additive fitness effects (h = ½). The top 1% theoretical quantile is enriched by ~30%, implying that ~2/10 of SNPs above the 99% threshold of the null are true positives.

Appendix D: Comparison of null model distributions for different metrics allele frequency differentiation between sexes

Three metrics have been used in empirical studies of allele frequency differentiation between the sexes: (1) between-sex $\hat{F}_{ST}$ (*e.g.*, Cheng and Kirkpatrick 2016; see Appendix A), (2) absolute allele frequency divergence between sexes, $|\hat{d}_{fm}|$ (*e.g.*, Berner 2019; Bissegger et al. 2019), and (3) the log odds ratio, $\hat{\mathcal{L}}$ (*e.g.*, Kasimatis et al. 2020 bioRχiv; see Appendix C). While each of these metrics has a distinct sampling distribution, and their null distributions differ in sensitivity to the minor allele frequency per SNP (MAF), the metrics share commonality in that all three are functions of estimated allele frequency differences between the sexes. Below, we briefly outline and compare the properties of their null distributions, and evaluate and compare their statistical power to detect SA SNPs with different minor allele frequencies.

Between-sex $\hat{F}_{ST}$

For large sample sizes, $\hat{F}_{ST}$ under the null (*i.e.*, no sex differences in selection) follows a chi-square distribution with one degree of freedom (see Appendix A). The expectation and the variance for $\hat{F}_{ST}$ are approximately:

$$\mathrm{E}[\hat{F}_{ST}] = \frac{1}{2n_H}$$

and

$$\mathrm{var}[\hat{F}_{ST}] = \frac{1}{2n_H^2}$$

and the kth quantile in the distribution of $\hat{F}_{ST}$ is:

$$\hat{F}_{ST(k)} = \frac{\left[\mathrm{erf}^{-1}\left(\frac{k}{100}\right)\right]^2}{n_H}$$

Note that the expectation, the variance, and quantiles of $\hat{F}_{ST}$ are all *independent* of minor allele frequencies in the population. Thus, the distribution of $\hat{F}_{ST}$ under the null model is constant across the spectrum of MAFs after SNPs with very rare alleles have been excluded from the analysis (*i.e.*, the approximations break down for polymorphic sites with MAF near zero; see Appendix A). Genome-wide predictions under the null model should, therefore, be relatively insensitive to details of the site frequency spectrum of the study population.

Absolute difference between female and male allele frequencies

The difference between female and male allele frequency estimates for a locus is:

$$\hat{d}_{fm} = \hat{p}_f - \hat{p}_m$$

which is well-approximated by a normal distribution provided the sample size of the study is large and the MAF at the locus is not too close to zero (see Appendix A). The expectation and variance for $\hat{d}_{fm}$ under the null hypothesis, conditioned on the SNP's MAF in the population (p), will be:

$$\mathrm{E}\left[\hat{d}_{fm}|p\right] = 0$$

$$\mathrm{var}\left[\hat{d}_{fm}|p\right] = p(1-p)\left(\frac{1}{n_f} + \frac{1}{n_m}\right) = \frac{2p(1-p)}{n_H}$$

(see Appendix A). The absolute allele frequency difference under the null will therefore follow a half-normal distribution with mean and variance of:

$$\mathrm{E}\left[\left|\hat{d}_{fm}\right|\middle|p\right] = 2\sqrt{\frac{p(1-p)}{\pi n_H}}$$

$$\mathrm{var}\left[\left|\hat{d}_{fm}\right|\middle|p\right] = \frac{2p(1-p)}{n_H}\left(1-\frac{2}{\pi}\right)$$

The kth quantile of the null distribution is:

$$\left|\hat{d}_{fm}\right|_{(k)} = 2\sqrt{\frac{p(1-p)}{n_H}}\,\mathrm{erf}^{-1}\left(\frac{k}{100}\right)$$

Note that each of these predictions is sensitive to the MAF of the locus and, consequently, the genome-wide null distribution for $\hat{d}_{fm}$ or for $\left|\hat{d}_{fm}\right|$ will be contingent on the focal population's site frequency spectrum, which will be unique to each study population.

Log Odds Ratio ($\hat{\mathcal{L}}$)

With a large sample size, and very low MAF sites excluded from the analysis (as before), the log odds ratio under the null model is well approximated by:

$$\hat{\mathcal{L}} = \log\left(\frac{\hat{p}_m(1-\hat{p}_f)}{(1-\hat{p}_m)\hat{p}_f}\right) = -\frac{(\hat{p}_f - \hat{p}_m)}{\hat{p}(1-\hat{p})}$$

(see Appendix C). Under the null model, $\hat{\mathcal{L}}$ will be approximately normal with mean and variance of:

$$\mathrm{E}\left[\hat{\mathcal{L}}|p\right] = 0$$

$$\mathrm{var}\left[\hat{\mathcal{L}}|p\right] = \frac{2}{n_H p(1-p)}$$

The distribution of the absolute value of $\hat{\mathcal{L}}$ ($|\hat{\mathcal{L}}|$) under the null will follow a half-normal distribution with mean and variance:

$$\mathrm{E}\left[|\hat{\mathcal{L}}|\,\middle|\,p\right] = \frac{2}{\sqrt{\pi n_H p(1-p)}}$$

$$\mathrm{var}\left[|\hat{\mathcal{L}}|\,\middle|\,p\right] = \frac{2}{n_H p(1-p)}\left(1 - \frac{2}{\pi}\right)$$

and the kth quantile of the null distribution will be:

$$|\hat{\mathcal{L}}|_{(k)} = \frac{2\,\mathrm{erf}^{-1}\left(\frac{k}{100}\right)}{\sqrt{n_H p(1-p)}}$$

Each of these predictions is sensitive to the MAF of the locus and, consequently, the genome-wide null distribution for $\hat{\mathcal{L}}$ or for $|\hat{\mathcal{L}}|$ will be contingent on the population's site frequency spectrum.

Evaluation of the metrics

The following figure shows simulated data for 50,000 non-singleton SNPs with minor allele frequencies uniformly distributed between ~0 to 0.5. Allele frequencies in the population were equal between the sexes, and 500 individuals of each sex were sampled and sequenced, resulting in sample sizes of $n_f = n_m = n_H = 1000$ gene sequences, per diploid locus, per sex. Each locus is polymorphic in both female and male samples, which excludes singleton polymorphisms from the analysis. Each of the three metrics of allele frequency differentiation was calculated from the simulated allele frequency estimates, with each grey dot showing a given metric's value for a single SNP. We compared the distribution of each metric relative to its appropriate null model. For F_{ST} estimates, we calculated the fraction of simulated SNPs where $\hat{F}_{ST}$ was greater than the 99% quantile defined by the null model approximations presented above. For the other two metrics—in which the null distribution either increases ($|\hat{d}_{fm}|$) or decreases ($|\hat{\mathcal{L}}|$) with MAF—we evaluated the proportion of SNPs that exceeded the 99% quantile defined by the SNP's MAF.

Across many simulation runs, we found that the proportion of SNPs above the appropriate 99% theoretical quantile was always identical between $\hat{F}_{ST}$ and $|\hat{d}_{fm}|$, which is unsurprising given that the theoretical distributions for both metrics are simple functions of the absolute difference between $\hat{p}_f$ and $\hat{p}_m$ (for the simulations shown in the figure below, 0.912% of SNPs for each metric are above the 99% threshold defined by the theoretical null model; individual simulation runs always yielded values near 1%, as expected). Roughly 1% of simulated $|\hat{\mathcal{L}}|$ values exceeded the 99% theoretical quantile for $|\hat{\mathcal{L}}|$ (0.988% for the simulation shown in the figure). Slight quantitative differences between the proportion of outliers in $|\hat{\mathcal{L}}|$ versus $\hat{F}_{ST}$ and $|\hat{d}_{fm}|$ reflects our use of an additional approximation in deriving the distribution of $|\hat{\mathcal{L}}|$ (simulated data relied on no such approximation).

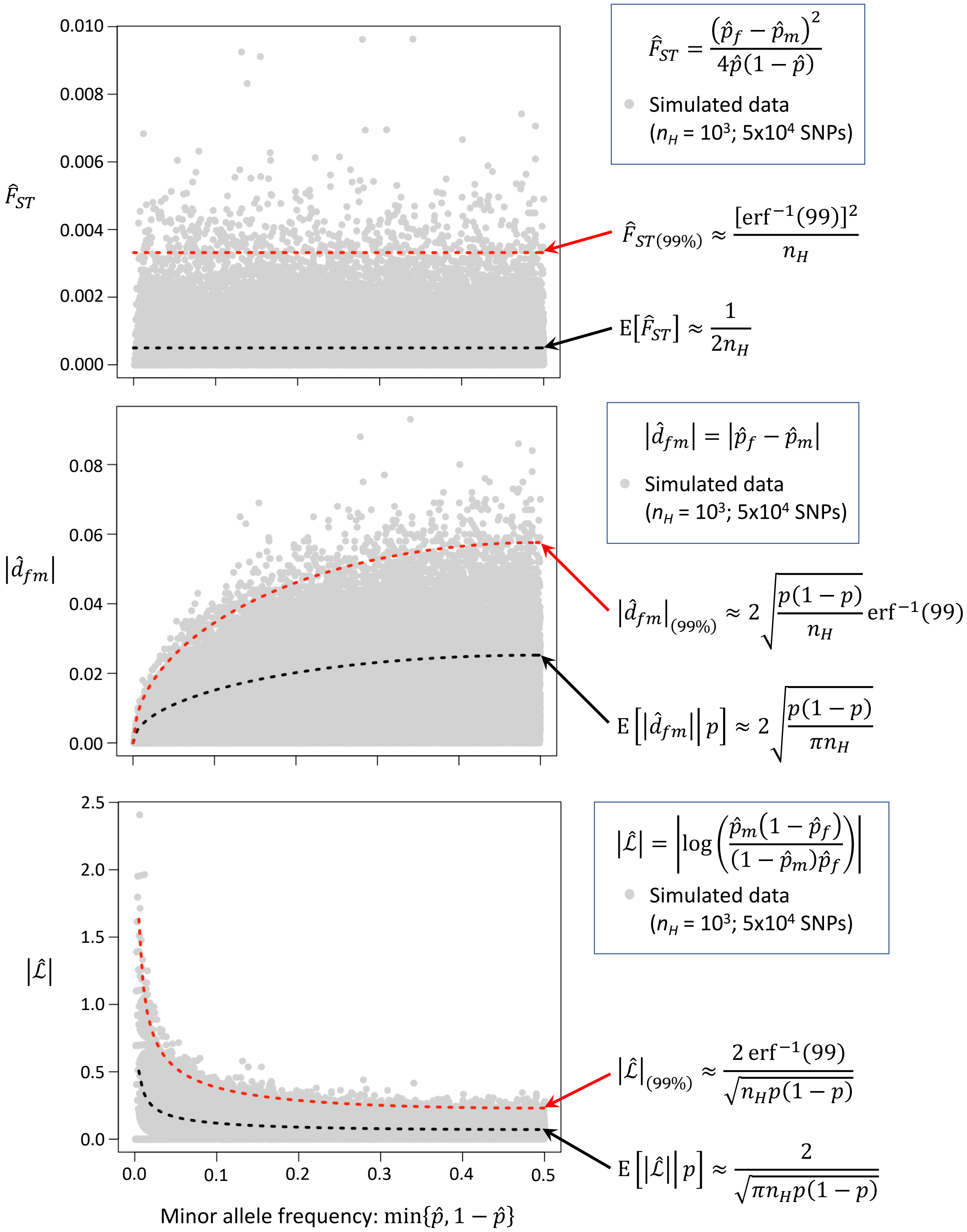

Statistical power to detect SA loci

The power to detect SA loci is roughly the same between the three metrics of allele frequency divergence between sexes, as shown in the following simulations. Simulations show the probability that $\hat{F}_{ST}$, $|\hat{d}_{fm}|$, or $|\hat{\mathcal{L}}|$ for an additive SA locus (*i.e.*, $h_f = h_m = ½$) is in the top 5% tail of the null distribution for the metric. The average cost to the sexes of inheriting the wrong SA allele is given by $\bar{s} = (t_f + |s_m|)/2$, where $|s_m|$ is the fitness cost to males of

being homozygous for the female-beneficial allele, and t_f is the cost to females of being homozygous for the male-beneficial allele (see Appendix A). Simulation results show the proportion of 10^5 simulated SA SNPs (per combination of n_H, p, and $\bar{s}$) that exhibits a greater between-sex divergence than the 95th percentile of the null distribution for the appropriate metric (95th percentile thresholds are based on the equations presented directly above). For each simulated SNP, the population is assumed to be at equilibrium for an additive SA locus with p representing the sex-averaged frequency of the female-beneficial allele at equilibrium; female and male allele frequencies, after selection, are $p_f = p + (p_f - p_m)/2$ and $p_m = p - (p_f - p_m)/2$, where the exact expression for $p_f - p_m$, at equilibrium, is presented in Appendix A.

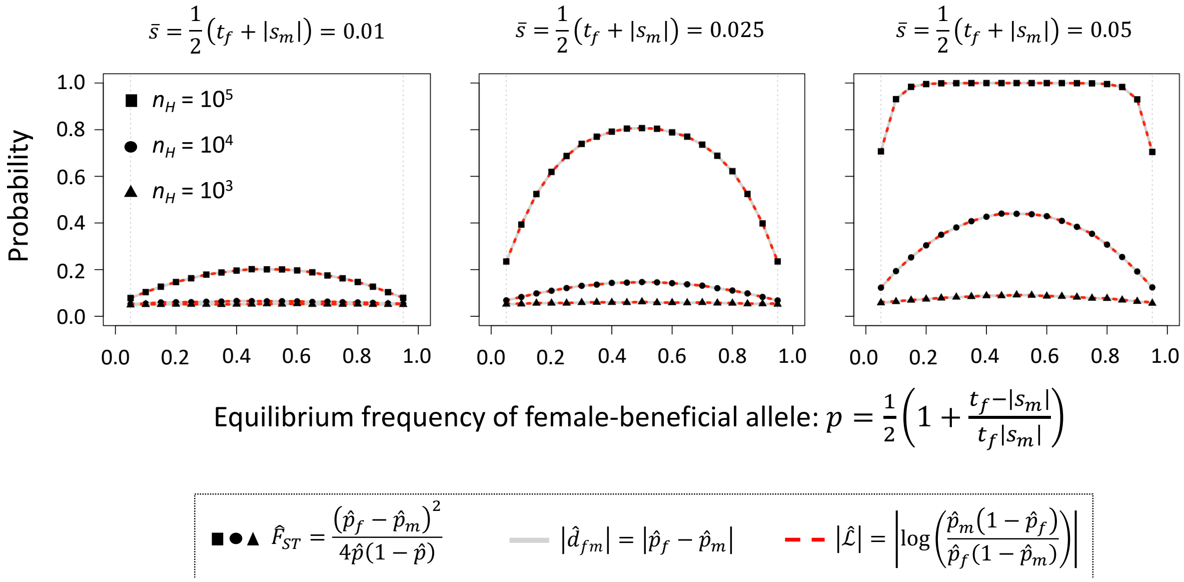

Supplementary References

Bishop, Y.M.M., Fienberg, S.E., and Holland, P.W. (1975). Section 14.6 in Discrete Multivariate Analysis. Cambridge, Massachusetts: MIT Press.

Hudson RR, Slatkin M, Maddison WP. 1992. Estimation of levels of gene flow from DNA sequence data. *Genetics* 132:583-589.

Morris JA, Gardner MJ. 1988. Calculating confidence intervals for relative risks (odds ratios) and standardised ratios and rates. *British Medical Journal* 296:1313-1316.

Nei M, Chakravarti A. 1977. Drift variances of F_{ST} and G_{ST} statistics obtained from a finite number of isolated populations. *Theor Pop Biol.* 11:307-325.

Wang W, Shan G. 2015. Exact Confidence Intervals for the Relative Risk and the Odds Ratio. *Biometrics* 71:985-995.

Weir BS, Cockerham CC. 1984. Estimating F-statistics for the analysis of population structure. *Evolution* 38:1358-1370.

Woolf, B. 1955. On estimating the relation between blood group and disease. Annals of Human Genetics 19, 251-253.
